# On a product-type operator between Hardy and *α*-Bloch spaces of the upper half-plane

**DOI:** 10.1186/s13660-018-1867-8

**Published:** 2018-10-10

**Authors:** Stevo Stević, Ajay K. Sharma

**Affiliations:** 1 Mathematical Institute of the Serbian Academy of Sciences, Beograd, Serbia; 2 Department of Medical Research, China Medical University Hospital, China Medical University, Taichung, Taiwan, Republic of China; 3 Department of Mathematics, Central University of Jammu, Jammu, India

**Keywords:** 47B38, 46E10, 30D45, Product-type operator, Hardy space, Bloch space, Boundedness, Compactness, Upper half-plane

## Abstract

Recently we have introduced a product-type operator and studied it on some spaces of analytic functions on the unit disc. Here we start investigating the operator on the space of analytic functions on the upper half-plane. We characterize the boundedness and compactness of the operator between Hardy and *α*-Bloch spaces on the domain.

## Introduction

Let ${\mathbb {D}}$ be the unit disc in the complex plane ${\mathbb{C}}$, $\Pi^{+}=\{z \in \mathbb{C} : \Im z > 0\}$ the upper half-plane in ${\mathbb {C}}$, and $\widehat{\Pi ^{+}}=\overline{\Pi^{+}} \cup \{\infty \}$. Let Ω be a domain in ${\mathbb {C}}$. We denote by $H(\Omega )$ the space of all analytic functions on Ω and by $S(\Omega )$ the class of all analytic self-maps of Ω.

For $0< p<\infty $, the *Hardy space* of $\Pi^{+}$, denoted by $H^{p}(\Pi^{+})$, consists of all $f\in H(\Pi^{+})$ such that

$$ \Vert f \Vert ^{p}_{H^{p}(\Pi^{+})} = \sup_{y > 0} \int_{-\infty }^{\infty } \bigl\vert f(x + iy) \bigr\vert ^{p} \,dx < \infty . $$

For $1 \leq p < \infty $, $H^{p}(\Pi^{+})$ is a Banach space.

Let $\alpha >0$. The *α-Bloch* or *Bloch-type* space on $\Pi^{+}$, denoted by $\mathcal{B}^{\alpha }(\Pi^{+})$, consists of all $f \in H(\Pi^{+})$ such that

1 $$\begin{aligned} \Vert f \Vert _{\mathcal{B}^{\alpha }( \Pi^{+})}:= \bigl\vert f(i) \bigr\vert + \sup _{z \in \Pi^{+}} ( \Im z)^{\alpha } \bigl\vert f'(z) \bigr\vert < \infty . \end{aligned}$$

With the norm (1), the *α*-Bloch space is a Banach space. For Bloch-type spaces on various domains and operators on them, see, for example, [1–13] and the references therein. For a natural extension of Bloch-type spaces and for Zygmud-type spaces, see [14–16].

For $\varphi \in S(\Omega )$, the *composition operator* $C_{\varphi }$ is the linear operator defined by

2 $$\begin{aligned} C_{\varphi }(f) (z)=(f \circ \varphi ) (z)\quad \text{for } f \in H( \Omega ). \end{aligned}$$

For $\psi \in H(\Omega )$, the *multiplication operator* $M_{\psi }$ is defined on $H(\Omega )$ by

3 $$\begin{aligned} M_{\psi }f(z) = \psi (z) f(z)\quad \text{for } f \in H(\Omega ). \end{aligned}$$

The product of these two operators

4 $$\begin{aligned} W_{\varphi , \psi }=M_{\psi }\circ C_{\varphi } \end{aligned}$$

is the so-called *weighted composition operator*.

By *D* we denote the *differentiation operator*, that is,

5 $$\begin{aligned} D f = f',\quad f \in H(\Omega ). \end{aligned}$$

These concrete operators, along with some integral-type ones, are among those considerably studied, during the last five decades, on spaces of analytic functions on various domains in ${\mathbb {C}}$ or domains in the complex-vector space ${\mathbb {C}}^{n}$. Majority of the papers on the operators are devoted to investigating them on spaces of analytic functions on ${\mathbb {D}}$. Much less papers consider the operators on spaces of analytic functions on other domains, including the upper half-plane. Even for some popular operators, such as the composition ones, up to the end of the previous century, there are no many papers on popular spaces such as $H^{p}(\Pi^{+})$ (see, e.g., [17–20] and the references therein). Hence, any new result on the spaces and operators on $\Pi^{+}$ is of some interest. Regarding some operators on the mentioned spaces, let us mention that some basic results on the boundedness of composition operators from $H^{p}(\Pi^{+})$ to the classical Bloch space $\mathcal{B}(\Pi^{+})$ can be found in note [21]. For related investigations of composition or weighted composition operators on some other spaces, see [14, 15, 22–25]. Let us mention that the behavior of composition operators on spaces of analytic functions in $\Pi^{+}$ is considerably different from the behavior of composition operators on spaces of analytic functions in $\mathbb{D}$. For example, every analytic self-map of $\mathbb{D}$ induces a bounded composition operator on the corresponding Hardy space, which is not always the case on the space $H^{p}(\Pi^{+})$ [17, 18].

From 1968 to 2005, experts more or less studied theoretic properties of only operators (2)–(5) and integral-type ones on spaces of analytic functions in terms of their symbols. The only product-type operator among (2)–(5) is the weighted composition operator. Since 2005, some experts started studying some other product-type operators. The first product-type operators different from weighted composition ones that attracted some attention were the products of composition and differentiation operators (see, e.g., [7, 11, 13, 26–29] and the references therein). Some generalizations of the products of composition and differentiation operators, containing iterated differentiation, have appeared soon after them (see [12, 30–33]). Around 2008, Li and Stević have initiated studying products of integral and composition operators, including in some cases the differentiation operator, on spaces of analytic functions on ${\mathbb {D}}$ (see, e.g., [34]), which have been considerably studied recently (see, e.g., [3, 35–40]). For some other results and related product-type operators, see, for example, [4–6, 35, 41–46].

To treat product-type operators consisting of exactly one composition, multiplication and differentiation operator in a unified manner, we have recently introduced a generalized operator and studied it on the weighted Bergman spaces (see [45] and [46]). See also papers [5, 42] on the operator on some other spaces of functions defined by

6 $$ T_{\psi_{1},\psi_{2} ,\varphi }f(z)=\psi_{1}(z)f\bigl(\varphi (z)\bigr)+\psi _{2}(z)f'\bigl(\varphi (z)\bigr),\quad f \in H( \mathbb{D}), $$

where $\psi_{1}, \psi_{2} \in H(\mathbb{D})$ and $\varphi \in S( \mathbb{D})$.

It is clear that operator (6) includes the composition, multiplication, differentiation, weighted composition, weighted differentiation composition, and many other concrete operators, including those in [6, 41, 43], which are obtained by some concrete choices of the symbols $\psi_{1}$, $\psi_{2}$, and *φ*. This is one of the reasons why the operator is of some importance for investigation.

So far the operator has not been considered between spaces of analytic functions on the upper half-plane. Here we start investigating the operator on such spaces by characterizing the boundedness and compactness of the operator between the Hardy and *α*-Bloch spaces on the domain, that is, of $T_{\psi_{1} , \psi_{2} , \varphi }:H^{p}(\Pi ^{+})\to \mathcal{B}^{\alpha }(\Pi^{+})$. We provide a complete characterization of the compactness. The paper can be regarded as a continuation of our investigations in [14, 15, 22–25].

Throughout this paper, constants are denoted by *C*; they are positive and not necessarily the same at each occurrence. The notation $A \asymp B$ means that $B \lesssim A \lesssim B$, where $A \lesssim B$ means that there is a positive constant *C* such that $A \leq C B$.

## Auxiliary results

First, we quote a point evaluation lemma, which is a folklore result (see, e.g., [15, Lemma 3]).

### Lemma 1

*Let* $p\in (0,\infty )$ *and* $n\in {\mathbb {N}}_{0}$. *Then*

$$ \bigl\vert f^{(n)}(z) \bigr\vert \le C\frac{ \Vert f \Vert _{H^{p}(\Pi^{+})}}{y^{n+\frac{1}{p}}} $$

*for some positive constant* $C=C(p,n)$ *independent of f*.

The following lemma can be found in [14, Lemma 2.1].

### Lemma 2

*Let* $p\in [1,\infty )$, $k\in {\mathbb {N}}_{0}$, $\zeta \in \Pi^{+}$, *and*

$$ f_{\zeta ,k}(z)=\frac{(\Im \zeta )^{k-\frac{1}{p}}}{(z-\overline{ \zeta })^{k}}. $$

*Then*

$$ \sup_{\zeta \in \Pi^{+}} \Vert f_{\zeta ,k} \Vert _{H^{p}(\Pi^{+})}\le \pi^{\frac{1}{p}}. $$

To deal with the compactness of the operator $T_{\psi_{1},\psi_{2}, \varphi }: H^{p}(\Pi^{+})\to \mathcal{B}^{\alpha }(\Pi^{+})$, in the lemma that follows, we give its characterization, which is typical for concrete operators between spaces of analytic functions. The thesis of Schwartz [47] is one of the first sources that presents such a characterization for the case of a concrete operator, more precisely, for a composition operator. It is interesting that the proof of our present lemma is more complicated than those of the corresponding lemmas that we have had so for (for example, the lemma in [24]). Hence we will present a detailed proof of the lemma.

We say that a sequence $(f_{n})_{n\in {\mathbb {N}}}$ in $H^{p}(\Pi^{+})$ converges weakly to zero if it is norm bounded in $H^{p}(\Pi^{+})$ and converges to zero on compacts of $\Pi^{+}$.

### Lemma 3

*Let* $p\ge 1$, $\alpha \in (0,+\infty )$, $\psi_{1},\psi_{2}\in H(\Pi^{+})$, *and* $\varphi \in S(\Pi^{+})$. *Then* $T_{\psi_{1},\psi_{2},\varphi }:H ^{p}(\Pi^{+})\rightarrow \mathcal{B}^{\alpha }( \Pi^{+})$ *is compact if and only if for any sequence* $(f_{n})_{n\in {\mathbb {N}}}$ *weakly convergent to zero*, *we have*

7 $$\begin{aligned} \lim_{n\rightarrow \infty } \Vert T_{\psi_{1},\psi_{2},\varphi } f_{n} \Vert _{\mathcal{B}^{\alpha }(\Pi^{+})}=0. \end{aligned}$$

### Proof

Let the operator be compact. Suppose that $(f_{n})_{n \in {\mathbb {N}}}$ weakly converges to zero. Then there are a subsequence $(f_{n_{k}})_{k\in {\mathbb {N}}}$ and $g\in \mathcal{B}^{\alpha }(\Pi^{+})$ such that

8 $$\begin{aligned} \lim_{k\to \infty } \Vert T_{\psi_{1},\psi_{2},\varphi } f_{n_{k}}-g \Vert _{ \mathcal{B}^{\alpha }(\Pi^{+})}=0. \end{aligned}$$

Let $K\subset \Pi^{+}$ be compact. Then

$$ d_{K}:=d\bigl(K,\partial \Pi^{+}\bigr)=\inf_{z\in K, x\in {\mathbb {R}}} \vert z-x \vert >0. $$

From this and from (1) we have

9 $$\begin{aligned} \bigl\vert T_{\psi_{1},\psi_{2},\varphi }f_{n_{k}}(i) - g(i) \bigr\vert \leq \Vert T_{\psi _{1},\psi_{2},\varphi }f_{n_{k}} - g \Vert _{\mathcal{B}^{\alpha }(\Pi^{+})} \end{aligned}$$

and

10 $$\begin{aligned} \sup_{z\in K} \bigl\vert \bigl(T_{\psi_{1},\psi_{2},\varphi }f_{n_{k}}(z) - g(z)\bigr)' \bigr\vert \leq \frac{1}{d_{K}} \Vert T_{\psi_{1},\psi_{2},\varphi }f_{n_{k}} - g \Vert _{\mathcal{B}^{\alpha }(\Pi^{+})}. \end{aligned}$$

Let $A\subset \Pi^{+}$ and $A_{\varepsilon }=\{z\in \Pi^{+}:d(z,K)\le \varepsilon \}$, where $\varepsilon >0$. Note that if *A* is compact, then for each $\varepsilon < d _{K}$, the set $A_{\varepsilon }$ is also a compact set as bounded and closed.

On the other hand, we have

11 $$\begin{aligned} f(z)=f(i)+ \int_{i}^{z}f'(\zeta )\,d\zeta \end{aligned}$$

for all $f\in H(\Pi^{+})$ and $z\in \Pi^{+}$.

Let $A\subset \Pi^{+}$ and

$$ A(i)=\bigl\{ w\in \Pi^{+}: \exists z\in A, w\in [i,z]\bigr\} . $$

Note that if *A* is compact, then $A(i)$ is also compact.

Hence from (11) we easily get

12 $$\begin{aligned} \bigl\vert f(z) \bigr\vert \le \bigl\vert f(i) \bigr\vert +\operatorname {diam}\bigl(K(i)\bigr)\sup_{w\in K(i)} \bigl\vert f'(w) \bigr\vert \end{aligned}$$

for each $z\in K$.

From (9), (10), and (12) it follows that

13 $$\begin{aligned} \sup_{z\in K} \bigl\vert T_{\psi_{1},\psi_{2},\varphi }f_{n_{k}}(z) - g(z) \bigr\vert & \le \bigl\vert (T_{\psi_{1},\psi_{2},\varphi }f_{n_{k}}(i) - g(i) \bigr\vert \\ &\quad {} +\operatorname {diam}\bigl(K(i)\bigr)\sup_{z\in K(i)} \bigl\vert \bigl(T_{\psi_{1},\psi_{2}, \varphi }f_{n_{k}}(z) - h(z)\bigr)' \bigr\vert \\ &\le \biggl( 1+\frac{\operatorname {diam}(K(i))}{d_{K(i)}} \biggr) \Vert T_{\psi_{1}, \psi_{2},\varphi }f_{n_{k}} - g \Vert _{\mathcal{B}^{\alpha }(\Pi^{+})}. \end{aligned}$$

From (8) and (13) it follows that

14 $$\begin{aligned} T_{\psi_{1},\psi_{2},\varphi }f_{n_{k}}(z) - g(z)\rightrightarrows 0, \quad k\to \infty , \end{aligned}$$

on each compact $K\in \Pi^{+}$.

Further, note that

$$ \widehat{M}_{j}:=\sup_{z\in K} \bigl\vert \psi_{j}(z) \bigr\vert < \infty ,\quad j=1,2, $$

for each compact *K*. The compactness of the set $\varphi (K)$ also implies that

$$ \max \bigl\{ f_{n_{k}}\bigl(\varphi (z)\bigr), f_{n_{k}}' \bigl(\varphi (z)\bigr)\bigr\} \rightrightarrows 0,\quad k\to \infty , $$

on each compact $K\in \Pi^{+}$.

From this, since

$$ \bigl\vert T_{\psi_{1},\psi_{2},\varphi }f_{n_{k}}\bigl(\varphi (z)\bigr) \bigr\vert \le \widehat{M}_{1} \sup_{w\in \varphi (K)} \bigl\vert f_{n_{k}}(w) \bigr\vert +\widehat{M}_{2}\sup_{w\in \varphi (K)} \bigl\vert f _{n_{k}}'(w) \bigr\vert , $$

it follows that

15 $$\begin{aligned} T_{\psi_{1},\psi_{2},\varphi }f_{n_{k}}(z)\rightrightarrows 0,\quad k \to \infty , \end{aligned}$$

on each compact $K\in \Pi^{+}$.

From (14) and (15) we obtain $g(z)=0$ for every $z\in \Pi^{+}$, since each $z\in \Pi^{+}$ lies in a compact subset of $\Pi^{+}$.

Using the fact in (8), it follows that

16 $$\begin{aligned} \lim_{k\to \infty } \Vert T_{\psi_{1},\psi_{2},\varphi } f_{n_{k}} \Vert _{ \mathcal{B}^{\alpha }(\Pi^{+})}=0. \end{aligned}$$

Such a procedure can be applied to any subsequence of $(f_{n})_{n \in {\mathbb {N}}}$, from which it follows that (7) holds.

Now assume that, for any sequence $(f_{n})_{n\in {\mathbb {N}}}$ weakly convergent to zero, we have $\lim_{n\rightarrow \infty } \Vert T_{\psi_{1}, \psi_{2},\varphi } f_{n} \Vert _{\mathcal{B}^{\alpha }(\Pi^{+})}=0$. Let $(\widehat{f}_{n})_{n\in {\mathbb {N}}}$ be a sequence of functions such that $M:=\sup_{n\in {\mathbb {N}}} \Vert \widehat{f}_{n} \Vert _{H^{p}(\Pi^{+})}<+\infty $. By Lemma 1 the sequence is uniformly bounded on compacts of $\Pi^{+}$, and consequently normal. Hence there are a subsequence $(\widehat{f}_{n _{k}})_{k\in {\mathbb {N}}}$ and $\widehat{f}\in H(\Pi^{+})$ such that

17 $$\begin{aligned} \widehat{f}_{n_{k}}(z) - \widehat{f}(z)\rightrightarrows 0,\quad k \to \infty , \end{aligned}$$

on each compact $K\in \Pi^{+}$. The Fatou lemma along with (17) implies $\Vert \widehat{f} \Vert _{H^{p}(\Pi^{+})} \leq M$. Hence, the sequence $(\widehat{f}_{n_{k}}-\widehat{f})_{k\in {\mathbb {N}}}$ weakly converges to zero, and consequently

$$ \lim_{k\to \infty } \Vert T_{\psi_{1},\psi_{2},\varphi }\widehat{f}_{n_{k}}-T _{\psi_{1},\psi_{2},\varphi }\widehat{f} \Vert _{\mathcal{B}^{\alpha }(\Pi^{+})}=0, $$

from which the compactness of the operator $T_{\psi_{1},\psi_{2}, \varphi }:H^{p}(\Pi^{+})\rightarrow \mathcal{B}^{\alpha }( \Pi^{+})$ follows. □

## Boundedness and compactness of $T_{\psi_{1},\psi_{2},\varphi}:H^{p}(\Pi^{+})\to\mathcal{B}^{\alpha}(\Pi^{+})$

In this section, we characterize the boundedness and compactness of the operator $T_{\psi_{1} , \psi_{2} , \varphi }:H^{p}(\Pi^{+})\to \mathcal{B}^{\alpha }(\Pi^{+})$. We also give upper and lower bounds for the norm of $T_{\psi_{1} , \psi_{2} , \varphi }$ acting between these spaces.

### Theorem 4

*Let* $1\leq p <\infty $, $\alpha >0$, $\psi_{1}, \psi_{2} \in H(\Pi ^{+})$ *and* $\varphi\in S(\Pi^{+})$. *Then* $T_{\psi_{1},\psi_{2},\varphi }: H^{p}(\Pi^{+})\to \mathcal{B}^{ \alpha }(\Pi^{+})$ *is bounded if and only if the following conditions are satisfied*:

$$\begin{aligned}& (\mathrm{i})\quad M_{1}= \sup_{z \in \Pi^{+}} \frac{(\Im z)^{\alpha }}{(\Im \varphi (z))^{1/p}} \bigl\vert \psi_{1}'(z) \bigr\vert < \infty ; \\& (\mathrm{ii})\quad M_{2} = \sup_{z \in \Pi^{+}} \frac{(\Im z)^{\alpha }}{(\Im \varphi (z))^{1 + 1/p}} \bigl\vert \psi_{1}(z) \varphi ' (z) + \psi_{2}'(z) \bigr\vert < \infty ; \\& (\mathrm{iii})\quad M_{3} = \sup_{z \in \Pi^{+}} \frac{(\Im z)^{\alpha }}{(\Im \varphi (z))^{2 + 1/p}} \bigl\vert \psi_{2}(z) \varphi ' (z) \bigr\vert < \infty . \end{aligned}$$

*Moreover*,

18 $$\begin{aligned} M_{1} + M_{2} + M_{3} & \lesssim \Vert T_{\psi_{1},\psi_{2},\varphi } \Vert _{ H^{p}(\Pi^{+})\to \mathcal{B}^{\alpha }(\Pi^{+})} \\ & \lesssim \frac{ \vert \psi_{1}(i) \vert }{(\Im \varphi (i))^{1/p}} + \frac{ \vert \psi_{2}(i) \vert }{(\Im \varphi (i))^{1 + 1/p}} + M_{1} + M_{2} + M_{3}. \end{aligned}$$

### Proof

First, suppose that conditions (i)–(iii) hold. Then by Lemma 1 we have

19 $$\begin{aligned} &(\Im z)^{\alpha } \bigl\vert (T_{\psi_{1},\psi_{2},\varphi } f)'(z) \bigr\vert \\ &\quad = (\Im z)^{\alpha } \bigl\vert \psi_{1}'(z)f \bigl(\varphi (z)\bigr)+\bigl(\psi_{1}(z)\varphi ' (z) + \psi_{2}'(z)\bigr)f'\bigl(\varphi (z)\bigr) + \psi_{2}(z)\varphi ' (z)f'' \bigl( \varphi (z)\bigr) \bigr\vert \\ & \quad \leq C \Vert f \Vert _{H^{p}(\Pi^{+})} \biggl(\frac{(\Im z)^{\alpha }}{( \Im \varphi (z))^{1/p}} \bigl\vert \psi_{1}'(z) \bigr\vert + \frac{(\Im z)^{\alpha }}{( \Im \varphi (z))^{1 + 1/p}} \bigl\vert \psi_{1}(z)\varphi ' (z) + \psi_{2}'(z) \bigr\vert \\ & \quad\quad {} + \frac{(\Im z)^{\alpha }}{(\Im \varphi (z))^{2 + 1/p}} \bigl\vert \psi _{2}(z)\varphi ' (z) \bigr\vert \biggr) \\ &\quad \leq C (M_{1} + M_{2} + M_{3}) \Vert f \Vert _{H^{p}(\Pi^{+})}. \end{aligned}$$

We also have

20 $$\begin{aligned} \bigl\vert T_{\psi_{1},\psi_{2},\varphi } f(i) \bigr\vert & = \bigl\vert \psi_{1}(i) f\bigl(\varphi (i)\bigr)+ \psi_{2}(i)f' \bigl(\varphi (i)\bigr) \bigr\vert \\ & \leq C \Vert f \Vert _{H^{p}(\Pi^{+})} \biggl(\frac{ \vert \psi_{1}(i) \vert }{(\Im \varphi (i))^{1/p}} + \frac{ \vert \psi_{2}(i) \vert }{(\Im \varphi (i))^{1 + 1/p}} \biggr). \end{aligned}$$

Combining (19) and (20), we have

$$\begin{aligned} \Vert T_{\psi_{1},\psi_{2},\varphi } f \Vert _{\mathcal{B}^{\alpha }(\Pi^{+}) } & \lesssim \biggl( \frac{ \vert \psi_{1}(i) \vert }{(\Im \varphi (i))^{1/p}} + \frac{ \vert \psi_{2}(i) \vert }{(\Im \varphi (i))^{1 + 1/p}} + M_{1} + M_{2} + M_{3} \biggr) \Vert f \Vert _{H^{p}(\Pi^{+})}, \end{aligned}$$

from which it follows that

21 $$\begin{aligned} \Vert T_{\psi_{1},\psi_{2},\varphi } \Vert _{H^{p}(\Pi^{+}) \to \mathcal{B} ^{\alpha }(\Pi^{+}) } & \lesssim \frac{ \vert \psi_{1}(i) \vert }{(\Im \varphi (i))^{1/p}} + \frac{ \vert \psi_{2}(i) \vert }{( \Im \varphi (i))^{1 + 1/p}} + M_{1} + M_{2} + M_{3}. \end{aligned}$$

Conversely, suppose that $T_{\psi_{1},\psi_{2},\varphi } : H^{p}(\Pi ^{+})\to \mathcal{B}^{\alpha }(\Pi^{+})$ is bounded. Consider the family of functions

$$ f_{w}(z) = \frac{(\Im w)^{2 - 1/p}}{\pi^{1/p}(z - \overline{w})^{2}} - 4i \frac{(\Im w)^{3 - 1/p}}{\pi^{1/p}(z - \overline{w})^{3}} - 4 \frac{( \Im w)^{4 - 1/p}}{\pi^{1/p}(z - \overline{w})^{4}}, $$

where $w\in \Pi^{+}$.

Since the functions $f_{w}$ are linear combinations of the functions in Lemma 2 (for $k=2,3,4$), from this by using the lemma it follows that

22 $$\begin{aligned} L_{1}:=\sup_{w\in \Pi^{+}} \Vert f_{w} \Vert _{H^{p}(\Pi^{+})}< \infty . \end{aligned}$$

We also have

$$\begin{aligned}& f'_{w}(z) = \frac{-2(\Im w)^{2 - 1/p}}{\pi^{1/p}(z - \overline{w})^{3}} + 12i \frac{( \Im w)^{3 - 1/p}}{\pi^{1/p}(z - \overline{w})^{4}}+16 \frac{(\Im w)^{4 - 1/p}}{\pi^{1/p}(z - \overline{w})^{5}}, \\& f''_{w}(z) = \frac{6(\Im w)^{2 - 1/p}}{\pi^{1/p}(z - \overline{w})^{4}} - 48i \frac{( \Im w)^{3 - 1/p}}{\pi^{1/p}(z - \overline{w})^{5}}-80 \frac{(\Im w)^{4 - 1/p}}{\pi^{1/p}(z - \overline{w})^{6}}, \end{aligned}$$

from which with some simple calculation we obtain

23 $$\begin{aligned} f_{w}(w) = 0,\quad\quad f'_{w}(w) = 0 \quad \text{and}\quad f''_{w}(w) = \frac{1}{8 \pi^{1/p}(\Im w)^{2 + 1/p}}. \end{aligned}$$

Since $T_{\psi_{1},\psi_{2},\varphi }: H^{p}(\Pi^{+})\to \mathcal{B} ^{\alpha }(\Pi^{+})$ is bounded, we have

$$\begin{aligned} \Vert T_{\psi_{1},\psi_{2},\varphi }f_{w} \Vert _{\mathcal{B}^{\alpha }(\Pi ^{+}) } &\leq \Vert T_{\psi_{1},\psi_{2},\varphi } \Vert _{H^{p}(\Pi^{+}) \to \mathcal{B}^{\alpha }(\Pi^{+}) } \Vert f_{w} \Vert _{H^{p}(\Pi^{+}) } \\ & \leq L_{1} \Vert T_{\psi_{1},\psi_{2},\varphi } \Vert _{H^{p}(\Pi^{+})\to \mathcal{B}^{\alpha }(\Pi^{+})} \end{aligned}$$

for every $w\in \Pi^{+}$.

Thus for each $\zeta \in \Pi^{+}$, we have

$$\begin{aligned} L_{1} \Vert T_{\psi_{1},\psi_{2},\varphi } \Vert _{H^{p}(\Pi^{+})\to \mathcal{B}^{\alpha }(\Pi^{+}) } & \geq (\Im \zeta )^{\alpha } \bigl\vert (T_{ \psi_{1},\psi_{2},\varphi }f_{\varphi (\zeta )})'( \zeta ) \bigr\vert \\ & = (\Im \zeta )^{\alpha } \bigl\vert \psi_{1}'( \zeta )f_{\varphi (\zeta )}\bigl(\varphi (\zeta )\bigr)+\bigl(\psi_{1}( \zeta )\varphi ' (\zeta )+ \psi_{2}'(\zeta )\bigr)f'_{\varphi (\zeta )} \bigl(\varphi (\zeta )\bigr) \\ &\quad {} + \psi _{2}(\zeta )\varphi ' (\zeta )f''_{\varphi (\zeta )}\bigl(\varphi (\zeta )\bigr) \bigr\vert \\ & = \frac{(\Im \zeta )^{\alpha } \vert \psi_{2}(\zeta )\varphi ' (\zeta ) \vert }{8 \pi^{1/p}(\Im \varphi (\zeta ))^{2 + 1/p}}. \end{aligned}$$

Since $\zeta \in \Pi^{+}$ is arbitrary, we have that

24 $$ M_{3} = \sup_{\zeta \in \Pi^{+}}\frac{(\Im \zeta )^{\alpha } \vert \psi_{2}(\zeta ) \varphi ' (\zeta ) \vert }{(\Im \varphi (\zeta ))^{2 + 1/p}} \leq 8\pi^{1/p}L _{1} \Vert T_{\psi_{1},\psi_{2},\varphi } \Vert _{H^{p}(\Pi^{+})\to \mathcal{B}^{\alpha }(\Pi^{+})}. $$

Now, consider the family of functions

$$ g_{w}(z) = 12 \frac{(\Im w)^{2 - 1/p}}{\pi^{1/p}(z - \overline{w})^{2}} - 32i \frac{( \Im w)^{3 - 1/p}}{\pi^{1/p}(z - \overline{w})^{3}} - 24 \frac{(\Im w)^{4 - 1/p}}{\pi^{1/p}(z - \overline{w})^{4}}. $$

Since the functions $g_{w}$ are also linear combinations of the functions in Lemma 2, we have $L_{2}:=\sup_{w\in \Pi^{+}} \Vert g_{w} \Vert _{H^{p}(\Pi^{+})} \le 1$.

We also have

$$\begin{aligned}& g'_{w}(z) = -24\frac{(\Im w)^{2-1/p}}{\pi^{1/p}(z-\overline{w})^{3}} + 96i \frac{(\Im w)^{3-1/p}}{\pi^{1/p}(z-\overline{w})^{4}} + 96 \frac{( \Im w)^{4 - 1/p}}{\pi^{1/p}(z - \overline{w})^{5}}, \\& g''_{w}(z) = 72\frac{(\Im w)^{2-1/p}}{\pi^{1/p}(z-\overline{w})^{4}} - 384i \frac{(\Im w)^{3-1/p}}{\pi^{1/p}(z-\overline{w})^{5}}-480 \frac{( \Im w)^{4 - 1/p}}{\pi^{1/p}(z - \overline{w})^{6}}, \end{aligned}$$

from which it follows that

$$ g'_{w}(w) = 0,\quad\quad g''_{w}(w) = 0, \quad \text{and}\quad g_{w}(w) =-\frac{1}{2 \pi^{1/p}(\Im w)^{1/p}}. $$

Since $T_{\psi_{1},\psi_{2},\varphi }: H^{p}(\Pi^{+})\to \mathcal{B} ^{\alpha }(\Pi^{+})$ is bounded, for each $\zeta \in \Pi^{+}$, we have

$$\begin{aligned} L_{2} \Vert T_{\psi_{1},\psi_{2},\varphi } \Vert _{H^{p}(\Pi^{+})\to \mathcal{B}^{\alpha }(\Pi^{+}) } &\ge \Vert T_{\psi_{1},\psi_{2},\varphi }g_{\varphi (\zeta )} \Vert _{\mathcal{B}^{\alpha }(\Pi^{+}) } \\ & = (\Im \zeta )^{\alpha } \bigl\vert \psi_{1}'( \zeta )g_{\varphi (\zeta )}\bigl(\varphi (\zeta )\bigr)+\bigl(\psi_{1}( \zeta )\varphi ' (\zeta ) + \psi_{2}'(\zeta ) \bigr)g'_{\varphi (\zeta )}\bigl(\varphi (\zeta )\bigr) \\ & \quad {}+ \psi_{2}( \zeta )\varphi ' (\zeta )g''_{\varphi (\zeta )} \bigl(\varphi (\zeta )\bigr) \bigr\vert \\ & = \frac{(\Im \zeta )^{\alpha } \vert \psi_{1}' (\zeta ) \vert }{2\pi^{1/p}( \Im \varphi (\zeta ))^{1/p}}. \end{aligned}$$

Since $\zeta \in \Pi^{+}$ is arbitrary, we have that

25 $$ M_{1} = \sup_{\zeta \in \Pi^{+}}\frac{(\Im \zeta )^{\alpha } \vert \psi_{1}' ( \zeta ) \vert }{(\Im \varphi (\zeta ))^{1/p}} \leq 2\pi^{1/p}L_{2} \Vert T_{\psi _{1},\psi_{2},\varphi } \Vert _{H^{p}(\Pi^{+})\to \mathcal{B}^{\alpha }(\Pi ^{+})}. $$

Now, consider the family of functions

$$ h_{w}(z) = 8\frac{(\Im w)^{2 - 1/p}}{\pi^{1/p}(z - \overline{w})^{2}} - 28i \frac{(\Im w)^{3 - 1/p}}{\pi^{1/p}(z - \overline{w})^{3}} - 24 \frac{( \Im w)^{4 - 1/p}}{\pi^{1/p}(z - \overline{w})^{4}}. $$

Once again proceeding as before, we can show that $\sup_{w\in \Pi^{+}} \Vert h_{w} \Vert _{H^{p}(\Pi^{+})} \lesssim 1$.

We have

$$\begin{aligned}& h'_{w}(z) = -16 \frac{(\Im w)^{2 - 1/p}}{\pi^{1/p}(z - \overline{w})^{3}} + 84i \frac{( \Im w)^{3 - 1/p}}{\pi^{1/p}(z - \overline{w})^{4}} + 96 \frac{(\Im w)^{4 - 1/p}}{\pi^{1/p}(z - \overline{w})^{5}}; \\& h''_{w}(z) = 48 \frac{(\Im w)^{2 - 1/p}}{\pi^{1/p}(z - \overline{w})^{4}} - 336i \frac{( \Im w)^{3 - 1/p}}{\pi^{1/p}(z - \overline{w})^{5}} - 480 \frac{( \Im w)^{4 - 1/p}}{\pi^{1/p}(z - \overline{w})^{6}}. \end{aligned}$$

Thus

$$ h_{w}(w) = 0,\quad\quad h''_{w}(w) = 0 \quad \text{and}\quad h'_{w}(w) = \frac{i}{4 \pi^{1/p}(\Im w)^{1+1/p}}. $$

Therefore by the boundedness of $T_{\psi_{1},\psi_{2},\varphi }: H ^{p}(\Pi^{+})\to \mathcal{B}^{\alpha }(\Pi^{+})$, for each $\zeta \in \Pi^{+}$, we have

$$\begin{aligned} L_{3} \Vert T_{\psi_{1},\psi_{2},\varphi } \Vert _{H^{p}(\Pi^{+})\to \mathcal{B}^{\alpha }(\Pi^{+}) } &\geq (\Im \zeta )^{\alpha } \bigl\vert \psi _{1}'(\zeta )h_{\varphi (\zeta )}\bigl(\varphi (\zeta )\bigr)+\bigl(\psi_{1}(\zeta ) \varphi ' (\zeta ) + \psi_{2}'(\zeta )\bigr)h'_{\varphi (\zeta )}\bigl(\varphi (\zeta )\bigr) \\ &\quad {} + \psi_{2}(\zeta ) \varphi ' (\zeta )h''_{\varphi (\zeta )} \bigl(\varphi (\zeta )\bigr) \bigr\vert \\ & = \frac{(\Im \zeta )^{\alpha } \vert \psi_{1}(\zeta )\varphi ' (\zeta ) + \psi_{2}'(\zeta ) \vert }{4\pi^{1/p}(\Im \varphi (\zeta ))^{1 +1/p}}, \end{aligned}$$

and consequently

26 $$ M_{2} = \sup_{\zeta \in \Pi^{+}}\frac{(\Im \zeta )^{\alpha } \vert \psi_{1}(\zeta ) \varphi ' (\zeta ) + \psi_{2}'(\zeta ) \vert }{(\Im \varphi (\zeta ))^{1 + 1/p}} \leq 4\pi^{1/p}L_{3} \Vert T_{\psi_{1},\psi_{2},\varphi } \Vert _{H^{p}( \Pi^{+})\to \mathcal{B}^{\alpha }(\Pi^{+})}. $$

Combining (24), (25), and (26), we have

$$ M_{1} + M_{2} + M_{3} \lesssim \Vert T_{\psi_{1}, \psi_{2},\varphi } \Vert _{H^{p}(\Pi^{+}) \to \mathcal{B}^{\alpha }(\Pi^{+})}, $$

finishing the proof of the theorem. □

### Corollary 5

*Let* $1\le p<\infty$, $\alpha >0$ *and* $\varphi \in S(\Pi^{+})$. *Then* $C_{\varphi }: H^{p}(\Pi^{+}) \to \mathcal{B}^{\alpha }(\Pi^{+})$ *is bounded if and only if*

$$ M_{4} = \sup_{z \in \Pi^{+}} \frac{(\Im z)^{\alpha }}{(\Im \varphi (z))^{1+1/p}} \bigl\vert \varphi '(z) \bigr\vert < \infty . $$

*Moreover*,

27 $$\begin{aligned} M_{4} \lesssim \Vert C_{\varphi } \Vert _{ H^{p}(\Pi^{+})\to \mathcal{B}^{ \alpha }(\Pi^{+})} \lesssim \frac{1}{(\Im \varphi (i))^{1/p}} + M_{4}. \end{aligned}$$

### Corollary 6

*Let* $1\le p<\infty$, $\alpha >0$, $\psi \in H(\Pi^{+})$ *and* $\varphi \in S(\Pi^{+})$. *Then* $M_{\psi } C_{\varphi }: H^{p}(\Pi^{+})\to \mathcal{B}^{\alpha }(\Pi ^{+})$ *is bounded if and only if*

$$\begin{aligned}& (\mathrm{i})\quad M_{5}= \sup_{z \in \Pi^{+}} \frac{(\Im z)^{\alpha }}{(\Im \varphi (z))^{1/p}} \bigl\vert \psi '(z) \bigr\vert < \infty , \\ & (\mathrm{ii})\quad M_{6} = \sup_{z \in \Pi^{+}} \frac{(\Im z)^{\alpha }}{(\Im \varphi (z))^{1 + 1/p}} \bigl\vert \psi (z)\varphi ' (z) \bigr\vert < \infty . \end{aligned}$$

*Moreover*,

28 $$\begin{aligned} M_{5} + M_{6} & \lesssim \Vert M_{\psi } C_{\varphi } \Vert _{ H^{p}(\Pi^{+}) \to \mathcal{B}^{\alpha }(\Pi^{+})} \lesssim \frac{ \vert \psi (i) \vert }{( \Im \varphi (i))^{1/p}} + M_{5} + M_{6}. \end{aligned}$$

### Corollary 7

*Let* $1\le p<\infty$, $\alpha >0$, $\psi \in H(\Pi^{+})$ *and* $\varphi \in S(\Pi^{+})$. *Then* $C_{\varphi } M_{\psi } : H^{p}(\Pi^{+})\to \mathcal{B}^{\alpha }(\Pi ^{+})$ *is bounded if and only if*

$$\begin{aligned}& (\mathrm{i})\quad M_{7}= \sup_{z \in \Pi^{+}} \frac{(\Im z)^{\alpha }}{(\Im \varphi (z))^{1/p}} \bigl\vert \psi '\bigl(\varphi (z)\bigr)\varphi ' (z) \bigr\vert < \infty , \\& (\mathrm{ii})\quad M_{8} = \sup_{z \in \Pi^{+}} \frac{(\Im z)^{\alpha }}{(\Im \varphi (z))^{1 + 1/p}} \bigl\vert \psi \bigl(\varphi (z)\bigr) \varphi ' (z) \bigr\vert < \infty . \end{aligned}$$

*Moreover*,

29 $$\begin{aligned} M_{7} + M_{8} & \lesssim \Vert C_{\varphi } M_{\psi } \Vert _{ H^{p}(\Pi^{+}) \to \mathcal{B}^{\alpha }(\Pi^{+})} \lesssim \frac{ \vert \psi (\varphi (i)) \vert }{( \Im \varphi (i))^{1/p}} + M_{7} + M_{8}. \end{aligned}$$

### Corollary 8

*Let* $1\le p<\infty$, $\alpha >0$ *and* $\varphi \in S(\Pi^{+})$. *Then* $C_{\varphi }D: H^{p}(\Pi^{+}) \to \mathcal{B}^{\alpha }(\Pi^{+})$ *is bounded if and only if*

$$ M_{9}= \sup_{z \in \Pi^{+}} \frac{(\Im z)^{\alpha }}{(\Im \varphi (z))^{2 + 1/p}} \bigl\vert \varphi ' (z) \bigr\vert < \infty . $$

*Moreover*,

30 $$\begin{aligned} M_{9} & \lesssim \Vert C_{\varphi }D \Vert _{ H^{p}(\Pi^{+})\to \mathcal{B} ^{\alpha }(\Pi^{+})} \lesssim \frac{1}{(\Im \varphi (i))^{1+1/p}} + M _{9}. \end{aligned}$$

### Corollary 9

*Let* $1\le p<\infty$, $\alpha >0$ *and* $\varphi \in S(\Pi^{+})$. *Then* $DC_{\varphi }: H^{p}(\Pi^{+}) \to \mathcal{B}^{\alpha }(\Pi^{+})$ *is bounded if and only if*

$$\begin{aligned}& (\mathrm{i})\quad M_{10} = \sup_{z \in \Pi^{+}} \frac{(\Im z)^{\alpha }}{(\Im \varphi (z))^{1 + 1/p}} \bigl\vert \varphi '' (z) \bigr\vert < \infty , \\& (\mathrm{ii})\quad M_{11} = \sup_{z \in \Pi^{+}} \frac{(\Im z)^{\alpha }}{(\Im \varphi (z))^{2 + 1/p}} \bigl\vert \varphi ' (z) \bigr\vert ^{2}< \infty . \end{aligned}$$

*Moreover*,

31 $$\begin{aligned} M_{10} + M_{11} \lesssim \Vert DC_{\varphi } \Vert _{ H^{p}(\Pi^{+})\to \mathcal{B}^{\alpha }(\Pi^{+})} \lesssim \frac{ \vert \varphi '(i) \vert }{( \Im \varphi (i))^{1+ 1/p}} + M_{10} + M_{11}. \end{aligned}$$

### Corollary 10

*Let* $1\le p<\infty$, $\alpha >0$, $\psi \in H(\Pi^{+})$ *and* $\varphi \in S(\Pi^{+})$. *Then* $M_{\psi }C_{\varphi }D: H^{p}(\Pi^{+})\to \mathcal{B}^{\alpha }(\Pi ^{+})$ *is bounded if and only if*

$$\begin{aligned}& (\mathrm{i})\quad M_{12} = \sup_{z \in \Pi^{+}} \frac{(\Im z)^{\alpha }}{(\Im \varphi (z))^{1 + 1/p}} \bigl\vert \psi ' (z) \bigr\vert < \infty , \\& (\mathrm{ii})\quad M_{13} = \sup_{z \in \Pi^{+}} \frac{(\Im z)^{\alpha }}{(\Im \varphi (z))^{2 + 1/p}} \bigl\vert \psi (z)\varphi ' (z) \bigr\vert < \infty . \end{aligned}$$

*Moreover*,

32 $$\begin{aligned} M_{12} + M_{13} \lesssim \Vert M_{\psi}C_{\varphi }D \Vert _{ H^{p}(\Pi^{+})\to \mathcal{B}^{\alpha }(\Pi^{+})} \lesssim \frac{ \vert \psi (i) \vert }{(\Im \varphi (i))^{1+1/p}} + M_{12} + M_{13}. \end{aligned}$$

### Corollary 11

*Let* $1\le p<\infty$, $\alpha >0$, $\psi \in H(\Pi^{+})$ *and* $\varphi \in S(\Pi^{+})$. *Then* $C_{\varphi }M_{\psi }D: H^{p}(\Pi^{+})\to \mathcal{B}^{\alpha }(\Pi ^{+})$ *is bounded if and only if*

$$\begin{aligned}& (\mathrm{i})\quad M_{14} = \sup_{z \in \Pi^{+}} \frac{(\Im z)^{\alpha }}{(\Im \varphi (z))^{1 + 1/p}} \bigl\vert \psi '\bigl(\varphi (z)\bigr)\varphi ' (z) \bigr\vert < \infty , \\& (\mathrm{ii})\quad M_{15} = \sup_{z \in \Pi^{+}} \frac{(\Im z)^{\alpha }}{(\Im \varphi (z))^{2 + 1/p}} \bigl\vert \psi \bigl(\varphi (z)\bigr) \varphi ' (z) \bigr\vert < \infty . \end{aligned}$$

*Moreover*,

33 $$\begin{aligned} M_{14} + M_{15} \lesssim \Vert C_{\varphi }M_{\psi }D \Vert _{ H^{p}(\Pi^{+}) \to \mathcal{B}^{\alpha }(\Pi^{+})} \lesssim \frac{ \vert \psi (\varphi (i)) \vert }{( \Im \varphi (i))^{1+1/p}} + M_{14} + M_{15}. \end{aligned}$$

### Corollary 12

*Let* $1\le p<\infty$, $\alpha >0$, $\psi \in H(\Pi^{+})$ *and* $\varphi \in S(\Pi^{+})$. *Then* $M_{\psi }D C_{\varphi }: H^{p}(\Pi^{+})\to \mathcal{B}^{\alpha }(\Pi ^{+})$ *is bounded if and only if*

$$\begin{aligned}& (\mathrm{i})\quad M_{16} = \sup_{z \in \Pi^{+}} \frac{(\Im z)^{\alpha }}{(\Im \varphi (z))^{1 + 1/p}} \bigl\vert \bigl(\psi \varphi ' \bigr)' (z) \bigr\vert < \infty , \\& (\mathrm{ii})\quad M_{17} = \sup_{z \in \Pi^{+}} \frac{(\Im z)^{\alpha }}{(\Im \varphi (z))^{2 + 1/p}} \bigl\vert \psi (z) \bigl(\varphi ' (z) \bigr)^{2} \bigr\vert < \infty . \end{aligned}$$

*Moreover*,

34 $$\begin{aligned} M_{16} + M_{17} \lesssim \Vert M_{\psi }D C_{\varphi } \Vert _{ H^{p}(\Pi^{+}) \to \mathcal{B}^{\alpha }(\Pi^{+})} \lesssim \frac{ \vert \psi (i)\varphi '(i) \vert }{( \Im \varphi (i))^{1+1/p}} + M_{16} + M_{17}. \end{aligned}$$

### Corollary 13

*Let* $1\le p<\infty$, $\alpha >0$, $\psi \in H(\Pi^{+})$ *and* $\varphi \in S(\Pi^{+})$. *Then* $D M_{\psi }C_{\varphi }: H^{p}(\Pi^{+})\to \mathcal{B}^{\alpha }(\Pi ^{+})$ *is bounded if and only if*

$$\begin{aligned}& (\mathrm{i})\quad M_{18} = \sup_{z \in \Pi^{+}} \frac{(\Im z)^{\alpha }}{(\Im \varphi (z))^{1/p}} \bigl\vert \psi ''(z) \bigr\vert < \infty , \\& (\mathrm{ii})\quad M_{19} = \sup_{z \in \Pi^{+}} \frac{(\Im z)^{\alpha }}{(\Im \varphi (z))^{1+ 1/p}} \bigl\vert 2\psi '(z) \varphi ' (z) + \psi(z) \varphi ''(z) \bigr\vert < \infty , \\& (\mathrm{iii})\quad M_{20} = \sup_{z \in \Pi^{+}} \frac{(\Im z)^{\alpha }}{(\Im \varphi (z))^{2 + 1/p}} \bigl\vert \psi (z) \bigl(\varphi ' (z) \bigr)^{2} \bigr\vert < \infty . \end{aligned}$$

*Moreover*,

$$\begin{aligned} M_{18} + M_{19} + M_{20} & \lesssim \Vert D M_{\psi }C_{\varphi } \Vert _{ H ^{p}(\Pi^{+})\to \mathcal{B}^{\alpha }(\Pi^{+})} \\ &\lesssim \frac{ \vert \psi '(i) \vert }{(\Im \varphi (i))^{1/p}} + \frac{ \vert \psi (i) \varphi '(i) \vert }{(\Im \varphi (i))^{1+1/p}} + M_{18} + M_{19} + M_{20}. \end{aligned}$$

### Corollary 14

*Let* $1\le p<\infty$, $\alpha >0$, $\psi \in H(\Pi^{+})$ *and* $\varphi \in S(\Pi^{+})$. *Then* $C_{\varphi }D M_{\psi }: H^{p}(\Pi^{+})\to \mathcal{B}^{\alpha }(\Pi ^{+})$ *is bounded if and only if*

$$\begin{aligned}& (\mathrm{i})\quad M_{21} = \sup_{z \in \Pi^{+}} \frac{(\Im z)^{\alpha }}{(\Im \varphi (z))^{1/p}} \bigl\vert \psi ''\bigl(\varphi (z) \bigr)\varphi ' (z) \bigr\vert < \infty , \\& (\mathrm{ii})\quad M_{22} = \sup_{z \in \Pi^{+}} \frac{(\Im z)^{\alpha }}{(\Im \varphi (z))^{1+ 1/p}} \bigl\vert \psi '\bigl(\varphi (z)\bigr)\varphi ' (z) \bigr\vert < \infty , \\& (\mathrm{iii})\quad M_{23} = \sup_{z \in \Pi^{+}} \frac{(\Im z)^{\alpha }}{(\Im \varphi (z))^{2 + 1/p}} \bigl\vert \psi \bigl(\varphi (z)\bigr) \varphi ' (z) \bigr\vert < \infty . \end{aligned}$$

*Moreover*,

$$\begin{aligned} M_{21} + M_{22} + M_{23} & \lesssim \Vert C_{\varphi }D M_{\psi } \Vert _{ H ^{p}(\Pi^{+})\to \mathcal{B}^{\alpha }(\Pi^{+})} \\ &\lesssim \frac{ \vert \psi '(\varphi (i)) \vert }{(\Im \varphi (i))^{1/p}} + \frac{ \vert \psi (\varphi (i)) \vert }{(\Im \varphi (i))^{1+1/p}} + M_{21} + M_{22} + M _{23}. \end{aligned}$$

### Corollary 15

*Let* $1\le p<\infty$, $\alpha >0$, $\psi \in H(\Pi^{+})$ *and* $\varphi \in S(\Pi^{+})$. *Then* $D C_{\varphi }M_{\psi }: H^{p}(\Pi^{+})\to \mathcal{B}^{\alpha }(\Pi ^{+})$ *is bounded if and only if*

$$\begin{aligned}& (\mathrm{i})\quad M_{24} = \sup_{z \in \Pi^{+}} \frac{(\Im z)^{\alpha }}{(\Im \varphi (z))^{1/p}} \bigl\vert \psi '\bigl(\varphi (z)\bigr)\varphi '' (z)+\psi ''\bigl(\varphi (z)\bigr) \bigl(\varphi ' (z)\bigr)^{2} \bigr\vert < \infty , \\& (\mathrm{ii})\quad M_{25} = \sup_{z \in \Pi^{+}} \frac{(\Im z)^{\alpha }}{(\Im \varphi (z))^{1+ 1/p}} \bigl\vert 2\psi '\bigl(\varphi (z)\bigr) \bigl( \varphi ' (z)\bigr)^{2} + \psi \bigl(\varphi (z)\bigr) \varphi '' (z) \bigr\vert < \infty , \\& (\mathrm{iii})\quad M_{26} = \sup_{z \in \Pi^{+}} \frac{(\Im z)^{\alpha }}{(\Im \varphi (z))^{2 + 1/p}} \bigl\vert \psi \bigl(\varphi (z)\bigr) \bigl( \varphi ' (z)\bigr)^{2} \bigr\vert < \infty . \end{aligned}$$

*Moreover*,

$$\begin{aligned} M_{24} + M_{25} + M_{26} & \lesssim \Vert D C_{\varphi }M_{\psi } \Vert _{ H ^{p}(\Pi^{+})\to \mathcal{B}^{\alpha }(\Pi^{+})} \\ & \lesssim \frac{ \vert \psi '(\varphi (i))\varphi '(i) \vert }{(\Im \varphi (i))^{1/p}} + \frac{ \vert \psi (\varphi (i))\varphi '(i) \vert }{(\Im \varphi (i))^{1+1/p}} + M_{24} + M_{25} + M_{26}. \end{aligned}$$

Recall that for a function *f* defined in $\Pi^{+}$, $\lim_{z \to \partial \widehat{\Pi ^{+}}}f(z)=0$ if and only if for every $\varepsilon >0$, there is a compact set $K\subset \Pi^{+}$ such that $\vert f(z) \vert <\varepsilon $ for $z\in \Pi^{+} \setminus K$.

The following result characterizes the compactness of the operator $T_{\psi_{1},\psi_{2},\varphi }: H^{p}(\Pi^{+})\to \mathcal{B}^{ \alpha }(\Pi^{+})$.

### Theorem 16

*Let* $1 \leq p < \infty $, $\alpha >0$, $\psi_{1},\psi_{2} \in H(\Pi^{+})$, *and* $\varphi \in S(\Pi^{+})$. *Then* $T_{\psi_{1},\psi_{2},\varphi }: H ^{p}(\Pi^{+})\to \mathcal{B}^{\alpha }(\Pi^{+})$ *is compact if and only if it is bounded and the following conditions are satisfied*:

$$\begin{aligned}& (\mathrm{i})\quad \lim_{\varphi (z)\to \widehat{\partial \Pi ^{+}}}\frac{(\Im z)^{ \alpha }}{(\Im \varphi (z))^{1/p}} \bigl\vert \psi_{1}'(z) \bigr\vert = 0, \\& (\mathrm{ii})\quad \lim_{\varphi (z)\to \widehat{\partial \Pi ^{+}}}\frac{(\Im z)^{ \alpha }}{(\Im \varphi (z))^{1 + 1/p}} \bigl\vert \psi_{1}(z)\varphi ' (z) + \psi _{2}'(z) \bigr\vert = 0, \\& (\mathrm{iii})\quad \lim_{\varphi (z)\to \widehat{\partial \Pi ^{+}}}\frac{(\Im z)^{ \alpha }}{(\Im \varphi (z))^{2 + 1/p}} \bigl\vert \psi_{2}(z)\varphi ' (z) \bigr\vert = 0. \end{aligned}$$

### Proof

Assume that the operator is bounded and conditions (i)–(iii) hold. Then by Theorem 4 the quantities $M_{j}$, $j=\overline{1,3}$, are finite, whereas from conditions (i)–(iii) we have that, for each $\varepsilon >0$, there exists a compact set *K* in $\Pi^{+}$ such that

35 $$\begin{aligned} &\frac{(\Im z)^{\alpha }}{(\Im \varphi (z))^{1/p}} \bigl\vert \psi_{1}'(z) \bigr\vert < \varepsilon , \end{aligned}$$

36 $$\begin{aligned} &\frac{(\Im z)^{\alpha }}{(\Im \varphi (z))^{1 + 1/p}} \bigl\vert \psi_{1}(z) \varphi ' (z) + \psi_{2}'(z) \bigr\vert < \varepsilon , \end{aligned}$$

37 $$\begin{aligned} &\frac{(\Im z)^{\alpha }}{(\Im \varphi (z))^{2 + 1/p}} \bigl\vert \psi_{2}(z) \varphi ' (z) \bigr\vert < \varepsilon , \end{aligned}$$

provided that $\varphi (z) \in \Pi^{+} \setminus K$.

Let $(f_{n})_{n\in {\mathbb {N}}}$ be a sequence in $H^{p}(\Pi^{+})$ weakly convergent to zero. Then, by inequalities (35)–(37) and Lemma 1, for every $z \in \Pi^{+}$ with $\varphi (z) \in \Pi^{+} \setminus K$, we have

$$\begin{aligned} &(\Im z)^{\alpha } \bigl\vert (T_{\psi_{1},\psi_{2},\varphi } f_{n})'(z) \bigr\vert \\ &\quad \leq (\Im z)^{\alpha } \bigl\vert \psi_{1}'(z)f_{n} \bigl(\varphi (z)\bigr)+\bigl(\psi_{1}(z) \varphi ' (z) + \psi_{2}'(z)\bigr)f'_{n}\bigl( \varphi (z)\bigr) + \psi_{2}(z)\varphi ' (z)f''_{n}\bigl(\varphi (z)\bigr) \bigr\vert \\ & \quad \leq C \biggl(\frac{(\Im z)^{\alpha }}{(\Im \varphi (z))^{1/p}} \bigl\vert \psi _{1}'(z) \bigr\vert + \frac{(\Im z)^{\alpha }}{(\Im \varphi (z))^{1 + 1/p}} \bigl\vert \psi _{1}(z)\varphi ' (z) + \psi_{2}'(z) \bigr\vert \\ & \quad \quad {} + \frac{(\Im z)^{\alpha }}{(\Im \varphi (z))^{2 + 1/p}} \bigl\vert \psi _{2}(z)\varphi ' (z) \bigr\vert \biggr) \Vert f_{n} \Vert _{H^{p}(\Pi^{+})} \\ & \quad < C \varepsilon . \end{aligned}$$

Since *K* is compact, $\varphi (K)$ is also compact. Hence

$$ M_{27}:=\sup_{w\in \varphi (z)}\Im w\in (0,\infty ). $$

Choose $n_{0} \in \mathbb{N}$ such that

38 $$\begin{aligned} \begin{gathered} \max_{z\in K} \bigl\vert f_{n}(z) \bigr\vert < \frac{\varepsilon }{M_{1}M_{27}^{1/p}}, \quad\quad \max_{z\in K} \bigl\vert f_{n}'(z) \bigr\vert < \frac{\varepsilon }{M_{2}M_{27}^{1/p+1}}, \\\max_{z\in K} \bigl\vert f_{n}''(z) \bigr\vert < \frac{\varepsilon }{M_{3}M_{27}^{1/p+2}},\end{gathered} \end{aligned}$$

and

39 $$\begin{aligned} \max \bigl\{ \bigl\vert f_{n}(i) \bigr\vert , \bigl\vert f_{n}'(i) \bigr\vert \bigr\} < \varepsilon \end{aligned}$$

for all $n \geq n_{0}$.

Then, by the preceding and (38), for every $z\in \Pi^{+}$ such that $\varphi (z)\in K$, we have

40 $$\begin{aligned} &(\Im z)^{\alpha } \bigl\vert \psi_{1}'(z)f_{n} \bigl(\varphi (z)\bigr)+\bigl(\psi_{1}(z)\varphi ' (z) + \psi_{2}'(z)\bigr)f'_{n}\bigl( \varphi (z)\bigr) + \psi_{2}(z)\varphi ' (z)f''_{n}\bigl( \varphi (z)\bigr) \bigr\vert \\ & \quad \leq (\Im z)^{\alpha } \bigl\vert \psi_{1}'(z) \bigr\vert \max_{w \in K } \bigl\vert f_{n}(w) \bigr\vert + ( \Im z)^{\alpha } \bigl\vert \psi_{1}(z)\varphi ' (z) + \psi_{2}'(z) \bigr\vert \max _{w \in K } \bigl\vert f'_{n}(w) \bigr\vert \\ &\quad \quad {} + (\Im z)^{\alpha } \bigl\vert \psi_{2}(z)\varphi ' (z) \bigr\vert \max_{w \in K } \bigl\vert f''_{n}(w) \bigr\vert \\ & \quad \leq M_{1}\bigl(\Im \varphi (z)\bigr)^{1/p} \max _{w \in K } \bigl\vert f_{n}(w) \bigr\vert + M _{2}\bigl(\Im \varphi (z)\bigr)^{1 + 1/p}\max _{w \in K } \bigl\vert f'_{n}(w) \bigr\vert \\ &\quad \quad {} +M_{3}\bigl(\Im \varphi (z)\bigr)^{2 +1/p}\max _{w \in K } \bigl\vert f''_{n}(w) \bigr\vert \\ &\quad \leq M_{1}\max_{w \in K } (\Im w)^{1/p} \max _{w \in K } \bigl\vert f_{n}(w) \bigr\vert + M_{2}\max_{w \in K } (\Im w)^{1 + 1/p}\max _{w \in K } \bigl\vert f'_{n}(w) \bigr\vert \\ &\quad \quad {} +M_{3}(\Im w)^{2 +1/p}\max_{w \in K } \bigl\vert f''_{n}(w) \bigr\vert \\ &\quad < 3\varepsilon . \end{aligned}$$

Further, using (39), we have

41 $$\begin{aligned} \bigl\vert T_{\psi_{1},\psi_{2},\varphi } f_{n}(i) \bigr\vert = \bigl\vert \psi_{1}(i) f_{n}\bigl(\varphi (i)\bigr)+ \psi_{2}(i)f'_{n}\bigl(\varphi (i)\bigr) \bigr\vert \le \bigl( \bigl\vert \psi_{1}(i) \bigr\vert + \bigl\vert \psi_{2}(i) \bigr\vert \bigr)\varepsilon \end{aligned}$$

for $n\ge n_{0}$.

From (40) and (41) it follows that

$$ \Vert T_{\psi_{1}, \psi_{2},\varphi } f_{n} \Vert _{\mathcal{B}^{\alpha }(\Pi ^{+})}< \bigl(3+ \bigl\vert \psi_{1}(i) \bigr\vert + \bigl\vert \psi_{2}(i) \bigr\vert \bigr)\varepsilon $$

for $n\ge n_{0}$.

Since $\varepsilon > 0$ is arbitrary, by Lemma 3 it follows that $T_{\psi_{1}, \psi_{2},\varphi }: H^{p}(\Pi^{+})\to \mathcal{B}^{ \alpha }(\Pi^{+})$ is compact.

Conversely, suppose that $T_{\psi_{1}, \psi_{2},\varphi }: H^{p}(\Pi ^{+})\to \mathcal{B}^{\alpha }(\Pi^{+})$ is compact. Then, clearly, it is bounded. Let $(z_{n})_{n\in {\mathbb {N}}}$ be a sequence in $\Pi^{+}$ such that $\varphi (z_{n})\to \widehat{\partial \Pi^{+}}$ as $n \to \infty $. If such a sequence does not exist, then conditions (i)–(iii) vacuously hold.

Let $w_{n} = \varphi (z_{n})$, $n\in {\mathbb {N}}$, and let the family of functions $(f_{n})_{n\in {\mathbb {N}}}$ be defined by

$$ f_{n}(z) = \frac{(\Im w_{n})^{2 - 1/p}}{\pi^{1/p}(z - \overline{w} _{n})^{2}} - 4i \frac{(\Im w_{n})^{3 - 1/p}}{\pi^{1/p}(z - \overline{w}_{n})^{3}} - 4 \frac{(\Im w_{n})^{4 - 1/p}}{\pi^{1/p}(z - \overline{w}_{n})^{4}}. $$

Then by using (22) and some simple estimates it is easy to see that $(f_{n})_{n\in {\mathbb {N}}}$ weakly converges to zero as $n\to \infty $ (including the case when $w_{n}\to\infty$ as $n\to\infty$). Hence

42 $$\begin{aligned} \lim_{n\to \infty } \Vert T_{\psi_{1}, \psi_{2},\varphi }f_{n} \Vert _{ \mathcal{B}^{\alpha }(\Pi^{+})}=0. \end{aligned}$$

The boundedness of $T_{\psi_{1}, \psi_{2},\varphi }: H^{p}(\Pi^{+}) \to \mathcal{B}^{\alpha }(\Pi^{+})$, together with (23), implies

43 $$\begin{aligned} \Vert T_{\psi_{1}, \psi_{2},\varphi }f_{n} \Vert _{\mathcal{B}^{\alpha }(\Pi ^{+})} &\geq (\Im z_{n})^{\alpha } \bigl\vert \psi_{1}'(z_{n})f_{n} \bigl(\varphi (z_{n})\bigr) +\bigl(\psi_{1}(z_{n}) \varphi ' (z_{n})+ \psi_{2}'(z_{n})\bigr)f'_{n} \bigl(\varphi (z_{n})\bigr) \\ &\quad {} + \psi_{2}(z_{n}) \varphi ' (z_{n})f''_{n} \bigl(\varphi (z_{n})\bigr) \bigr\vert \\ & = \frac{(\Im z_{n})^{\alpha }}{8\pi^{1/p}(\Im \varphi (z_{n}))^{2 + 1/p}} \bigl\vert \psi_{2}(z_{n})\varphi ' (z_{n}) \bigr\vert . \end{aligned}$$

From (42) and (43) we obtain

44 $$\begin{aligned} \lim_{\varphi (z_{n})\to \widehat{\partial \Pi ^{+}}}\frac{(\Im z_{n})^{ \alpha }}{(\Im \varphi (z_{n}))^{2 + 1/p}} \bigl\vert \psi_{2}(z_{n})\varphi ' (z _{n}) \bigr\vert = 0, \end{aligned}$$

from which it follows that condition (i) holds.

By considering families of functions

$$ g_{n}(z) = 12\frac{(\Im w_{n})^{2 - 1/p}}{\pi^{1/p}(z - \overline{w} _{n})^{2}} - 32i \frac{(\Im w_{n})^{3 - 1/p}}{\pi^{1/p}(z - \overline{w}_{n})^{3}} - 24 \frac{(\Im w_{n})^{4 - 1/p}}{\pi^{1/p}(z - \overline{w}_{n})^{4}} $$

and

$$ h_{n}(z) = 8\frac{(\Im w_{n})^{2 - 1/p}}{\pi^{1/p}(z - \overline{w} _{n})^{2}} - 28i \frac{(\Im w_{n})^{3 - 1/p}}{\pi^{1/p}(z - \overline{w}_{n})^{3}} - 24 \frac{(\Im w_{n})^{4 - 1/p}}{\pi^{1/p}(z - \overline{w}_{n})^{4}} $$

and proceeding as before, we can similarly show that

45 $$\begin{aligned} \lim_{\varphi (z_{n})\to \widehat{\partial \Pi ^{+}}}\frac{(\Im z_{n})^{ \alpha }}{(\Im \varphi (z_{n}))^{1 + 1/p}} \bigl\vert \psi_{1}(z_{n})\varphi ' (z _{n}) + \psi_{2}'(z_{n}) \bigr\vert = 0 \end{aligned}$$

and

46 $$\begin{aligned} \lim_{\varphi (z_{n})\to \widehat{\partial \Pi ^{+}}}\frac{(\Im z_{n})^{ \alpha }}{(\Im \varphi (z_{n}))^{1/p}} \bigl\vert \psi_{1}'(z_{n}) \bigr\vert = 0, \end{aligned}$$

from which it follows that conditions (ii) and (iii) hold, respectively. □

### Corollary 17

*Let* $1\le p<\infty$, $\alpha >0$ *and* $\varphi \in S(\Pi^{+})$. *Then* $C_{\varphi }: H^{p}(\Pi^{+}) \to \mathcal{B}^{\alpha }(\Pi^{+})$ *is compact if and only if it is bounded and*

$$ \lim_{\varphi (z)\to \widehat{\partial \Pi ^{+}}}\frac{(\Im z)^{ \alpha }}{(\Im \varphi (z))^{1+1/p}} \bigl\vert \varphi '(z) \bigr\vert = 0. $$

### Corollary 18

*Let* $1\le p<\infty$, $\alpha >0$, $\psi \in H(\Pi^{+})$ *and* $\varphi \in S(\Pi^{+})$. *Then* $M_{\psi}C_{\varphi}: H^{p}(\Pi^{+})\to \mathcal{B}^{\alpha }(\Pi^{+})$ *is compact if and only if it is bounded and*

$$\begin{aligned}& (\mathrm{i})\quad \lim_{\varphi (z)\to \widehat{\partial \Pi ^{+}}}\frac{(\Im z)^{ \alpha }}{(\Im \varphi (z))^{1/p}} \bigl\vert \psi '(z) \bigr\vert = 0, \\& (\mathrm{ii})\quad \lim_{\varphi (z)\to \widehat{\partial \Pi ^{+}}}\frac{(\Im z)^{ \alpha }}{(\Im \varphi (z))^{1 + 1/p}} \bigl\vert \psi (z)\varphi ' (z) \bigr\vert = 0. \end{aligned}$$

### Corollary 19

*Let* $1\le p<\infty$, $\alpha >0$, $\psi \in H(\Pi^{+})$ *and* $\varphi \in S(\Pi^{+})$. *Then* $C_{\varphi } M_{\psi } : H^{p}(\Pi^{+})\to \mathcal{B}^{\alpha }(\Pi ^{+})$ *is compact if and only if it is bounded and*

$$\begin{aligned}& (\mathrm{i})\quad \lim_{\varphi (z)\to \widehat{\partial \Pi ^{+}}}\frac{(\Im z)^{ \alpha }}{(\Im \varphi (z))^{1/p}} \bigl\vert \psi '\bigl(\varphi (z)\bigr)\varphi ' (z) \bigr\vert =0, \\& (\mathrm{ii})\quad \lim_{\varphi (z)\to \widehat{\partial \Pi ^{+}}}\frac{(\Im z)^{ \alpha }}{(\Im \varphi (z))^{1 + 1/p}} \bigl\vert \psi \bigl(\varphi (z)\bigr)\varphi ' (z) \bigr\vert =0. \end{aligned}$$

### Corollary 20

*Let* $1\le p<\infty$, $\alpha >0$ *and* $\varphi \in S(\Pi^{+})$. *Then* $C_{\varphi }D: H^{p}(\Pi^{+}) \to \mathcal{B}^{\alpha }(\Pi^{+})$ *is compact if and only if it is bounded and*

$$ \lim_{\varphi (z)\to \widehat{\partial \Pi ^{+}}}\frac{(\Im z)^{ \alpha }}{(\Im \varphi (z))^{2 + 1/p}} \bigl\vert \varphi ' (z) \bigr\vert = 0. $$

### Corollary 21

*Let* $1\le p<\infty$, $\alpha >0$ *and* $\varphi \in S(\Pi^{+})$. *Then* $DC_{\varphi }: H^{p}(\Pi^{+}) \to \mathcal{B}^{\alpha }(\Pi^{+})$ *is compact if and only if it is bounded and*

$$\begin{aligned}& (\mathrm{i})\quad \lim_{\varphi (z)\to \widehat{\partial \Pi ^{+}}}\frac{(\Im z)^{ \alpha }}{(\Im \varphi (z))^{1 + 1/p}} \bigl\vert \varphi '' (z) \bigr\vert = 0, \\& (\mathrm{ii})\quad \lim_{\varphi (z)\to \widehat{\partial \Pi ^{+}}}\frac{(\Im z)^{ \alpha }}{(\Im \varphi (z))^{2 + 1/p}} \bigl\vert \varphi ' (z) \bigr\vert ^{2} = 0. \end{aligned}$$

### Corollary 22

*Let* $1\le p<\infty$, $\alpha >0$, $\psi \in H(\Pi^{+})$ *and* $\varphi \in S(\Pi^{+})$. *Then* $M_{\psi }C_{\varphi }D: H^{p}(\Pi^{+})\to \mathcal{B}^{\alpha }(\Pi ^{+})$ *is compact if and only if it is bounded and*

$$\begin{aligned}& (\mathrm{i})\quad \lim_{\varphi (z)\to \widehat{\partial \Pi ^{+}}}\frac{(\Im z)^{ \alpha }}{(\Im \varphi (z))^{1 + 1/p}} \bigl\vert \psi ' (z) \bigr\vert = 0, \\& (\mathrm{ii})\quad \lim_{\varphi (z)\to \widehat{\partial \Pi ^{+}}}\frac{(\Im z)^{ \alpha }}{(\Im \varphi (z))^{2 + 1/p}} \bigl\vert \psi (z)\varphi ' (z) \bigr\vert = 0. \end{aligned}$$

### Corollary 23

*Let* $1\le p<\infty$, $\alpha >0$, $\psi \in H(\Pi^{+})$ *and* $\varphi \in S(\Pi^{+})$. *Then* $C_{\varphi }M_{\psi }D: H^{p}(\Pi^{+})\to \mathcal{B}^{\alpha }(\Pi ^{+})$ *is compact if and only if it is bounded and*

$$\begin{aligned}& (\mathrm{i})\quad \lim_{\varphi (z)\to \widehat{\partial \Pi ^{+}}}\frac{(\Im z)^{ \alpha }}{(\Im \varphi (z))^{1 + 1/p}} \bigl\vert \psi '\bigl(\varphi (z)\bigr)\varphi ' (z) \bigr\vert = 0, \\& (\mathrm{ii})\quad \lim_{\varphi (z)\to \widehat{\partial \Pi ^{+}}} \frac{(\Im z)^{ \alpha }}{(\Im \varphi (z))^{2 + 1/p}} \bigl\vert \psi \bigl(\varphi (z)\bigr)\varphi ' (z) \bigr\vert = 0. \end{aligned}$$

### Corollary 24

*Let* $1\le p<\infty$, $\alpha >0$, $\psi \in H(\Pi^{+})$ *and* $\varphi \in S(\Pi^{+})$. *Then* $M_{\psi }D C_{\varphi }: H^{p}(\Pi^{+})\to \mathcal{B}^{\alpha }(\Pi ^{+})$ *is compact if and only if it is bounded and*

$$\begin{aligned}& (\mathrm{i})\quad \lim_{\varphi (z)\to \widehat{\partial \Pi ^{+}}}\frac{(\Im z)^{ \alpha }}{(\Im \varphi (z))^{1 + 1/p}} \bigl\vert \bigl(\psi \varphi '\bigr)' (z) \bigr\vert = 0, \\& (\mathrm{ii})\quad \lim_{\varphi (z)\to \widehat{\partial \Pi ^{+}}} \frac{(\Im z)^{ \alpha }}{(\Im \varphi (z))^{2 + 1/p}} \bigl\vert \psi (z) \bigl(\varphi ' (z)\bigr)^{2} \bigr\vert = 0. \end{aligned}$$

### Corollary 25

*Let* $1\le p<\infty$, $\alpha >0$, $\psi \in H(\Pi^{+})$ *and* $\varphi \in S(\Pi^{+})$. *Then* $D M_{\psi }C_{\varphi }: H^{p}(\Pi^{+})\to \mathcal{B}^{\alpha }(\Pi ^{+})$ *is compact if and only if it is bounded and*

$$\begin{aligned}& (\mathrm{i})\quad \lim_{\varphi (z)\to \widehat{\partial \Pi ^{+}}} \frac{(\Im z)^{ \alpha }}{(\Im \varphi (z))^{1/p}} \bigl\vert \psi ''(z) \bigr\vert = 0, \\& (\mathrm{ii})\quad \lim_{\varphi (z)\to \widehat{\partial \Pi ^{+}}} \frac{(\Im z)^{\alpha }}{(\Im \varphi (z))^{1+ 1/p}} \bigl\vert 2\psi '(z) \varphi ' (z) +\psi(z) \varphi ''(z) \bigr\vert = 0, \\& (\mathrm{iii})\quad \lim_{\varphi (z)\to \widehat{\partial \Pi ^{+}}} \frac{(\Im z)^{\alpha }}{(\Im \varphi (z))^{2 + 1/p}} \bigl\vert \psi (z) \bigl(\varphi ' (z) \bigr)^{2} \bigr\vert = 0. \end{aligned}$$

### Corollary 26

*Let* $1\le p<\infty$, $\alpha >0$, $\psi \in H(\Pi^{+})$ *and* $\varphi \in S(\Pi^{+})$. *Then* $C_{\varphi }D M_{\psi }: H^{p}(\Pi^{+})\to \mathcal{B}^{\alpha }(\Pi ^{+})$ *is compact if and only if it is bounded and*

$$\begin{aligned}& (\mathrm{i})\quad \lim_{\varphi (z)\to \widehat{\partial \Pi ^{+}}}\frac{(\Im z)^{ \alpha }}{(\Im \varphi (z))^{1/p}} \bigl\vert \psi ''\bigl(\varphi (z)\bigr)\varphi ' (z) \bigr\vert = 0, \\& (\mathrm{ii})\quad \lim_{\varphi (z)\to \widehat{\partial \Pi ^{+}}}\frac{(\Im z)^{ \alpha }}{(\Im \varphi (z))^{1 + 1/p}} \bigl\vert \psi '\bigl(\varphi (z)\bigr)\varphi ' (z) \bigr\vert = 0, \\& (\mathrm{iii})\quad \lim_{\varphi (z)\to \widehat{\partial \Pi ^{+}}}\frac{(\Im z)^{ \alpha }}{(\Im \varphi (z))^{2 + 1/p}} \bigl\vert \psi \bigl(\varphi (z)\bigr)\varphi ' (z) \bigr\vert = 0. \end{aligned}$$

### Corollary 27

*Let* $1\le p<\infty$, $\alpha >0$, $\psi \in H(\Pi^{+})$ *and* $\varphi \in S(\Pi^{+})$. *Then* $D C_{\varphi }M_{\psi }: H^{p}(\Pi^{+})\to \mathcal{B}^{\alpha }(\Pi ^{+})$ *is compact if and only if it is bounded and*

$$\begin{aligned}& (\mathrm{i})\quad \lim_{\varphi (z)\to \widehat{\partial \Pi ^{+}}}\frac{(\Im z)^{ \alpha }}{(\Im \varphi (z))^{1/p}} \bigl\vert \psi '\bigl(\varphi (z)\bigr)\varphi ''(z)+ \psi ''\bigl(\varphi (z)\bigr) \bigl(\varphi ' (z)\bigr)^{2} \bigr\vert = 0, \\& (\mathrm{ii})\quad \lim_{\varphi (z)\to \widehat{\partial \Pi ^{+}}}\frac{(\Im z)^{ \alpha }}{(\Im \varphi (z))^{1 + 1/p}} \bigl\vert 2\psi '\bigl(\varphi (z)\bigr) \bigl(\varphi ' (z) \bigr)^{2} + \psi \bigl(\varphi (z)\bigr)\varphi '' (z) \bigr\vert = 0, \\& (\mathrm{iii})\quad \lim_{\varphi (z)\to \widehat{\partial \Pi ^{+}}}\frac{(\Im z)^{ \alpha }}{(\Im \varphi (z))^{2 + 1/p}} \bigl\vert \psi \bigl(\varphi (z)\bigr) \bigl(\varphi ' (z) \bigr)^{2} \bigr\vert = 0. \end{aligned}$$
